# Informal payments in public hospitals in Malawi: the case of Kamuzu Central Hospital

**DOI:** 10.1186/s41256-021-00225-z

**Published:** 2021-11-24

**Authors:** Annette Mphande-Namangale, Isabel Kazanga-Chiumia

**Affiliations:** 1 University of Malawi, College of Medicine, School of Public Health, Blantyre, Malawi; 2 University of Malawi, College of Medicine, Department of Health Systems and Policy, Lilongwe, Malawi

**Keywords:** Malawi, Informal payments

## Abstract

**Background:**

Informal payments in public health facilities act as a barrier to accessing quality health services, especially for poor people. This research aimed to investigate informal payments for health care services at Kamuzu Central Hospital (KCH), a public referral hospital in Malawi. Results of this study provide evidence on the prevalence and influencing factors of informal payments for health care so that relevant policies and strategies may be developed to address this problem.

**Methods:**

This study employed a mixed methods research design. The quantitative study had a sample size of 295 patients and guardians. The qualitative study had 7 key informant interviews (with health workers, health managers and policy makers) and 3 focus group discussions (FGDs) with guardians. Each FGD included 10 participants. Thus, in total, the qualitative sample comprised 52 participants. Quantitative data was analyzed using Excel and STATA. Qualitative data was analyzed using a thematic content analysis approach.

**Results:**

80% of patients and guardians had knowledge of informal payments. Approximately 47% of respondents admitted making informal payments to access health services, and 87% of informal payments were made at the request of a health worker. Lack of knowledge, fear and desperation among patients and guardians, low salaries of health workers, and lack of effective disciplinary measures, were reported as key factors influencing informal payments. Regression analysis results showed that occupation and gender were the main determinants of informal payments.

**Conclusions:**

Informal payments exacerbate inequality in access to free public health services. Particularly, poor people have limited access to health services when informal payments are demanded. This practice is unethical and infringes on people’s rights to universal access to health care. There is a need to strengthen the public health care system by formulating deliberate policies that will deter informal payments in Malawi.

## Background

Equitable access to health services is a fundamental requirement in the provision of health care. Public health services in Malawi are provided for free to all Malawians through the Essential Health Package (EHP) to promote universal access to health care [1]. However, since the majority of the population in Malawi (above 60%) lives on less than US$1.25 per day [2], informal payments act as a barrier limiting people’s access to health care services and violate the right to health care access by poverty-stricken patients [3]. An informal payment is any direct cash or non-cash payment that, in addition to the specified contribution, is provided by patients or their relatives to health care providers for services that the patients have legally been entitled to receive [4]. Other authors have also defined informal payments as “the use of public office for private gains” [5]. Health sector-related researchers often use the concept of “informal payments” as a term for the exchange of money, gifts or services between patients or their families and health care personnel [6].

There is growing evidence that many low-income countries have limited access to quality health care services due to demands for payments for services that should not be paid for [7]. The challenge with informal payments is that they are shrouded in secrecy and therefore are difficult to document [8]. These “under-the-table” payments motivate health workers and can also influence physicians regarding which health care services to provide to which patients [9]. In general, informal payments in public hospitals are seen by most as morally undesirable and unethical [4], and they can result in low-quality service in terms of time and services [6]. Citizens are not satisfied with their health care service, and they lose trust if they are made to pay for services that are meant to be free [10]. Nevertheless, some authors have suggested that poor working conditions and sociocultural environments force health workers to accept informal payments [11].

Informal payments are an important barrier to health care utilization for low-income patients [12], as corrupt health workers may create a sense of panic among people who are already suffering both physically and financially–by indicating, for example, that there is a drug or staff shortage and that they therefore have to pay to receive better service. Equity is also decreased because informal payments deter poor people from utilizing health services, and they are forced to incur unnecessary costs [13].

Informal payments infringe on people’s right to quality health care as stipulated by the World Health Organization. Informal payments may eventually lead to corruption, in which case those who cannot afford to pay fail to access health services or delay to seek health care. This takes away the right of an individual to get quality health services and this is therefore identified as human right violation, identified under international law [14]. Violations or lack of attention to human rights can have serious health consequences, and act as a barrier to health services, and contribute to poor quality care [15].

Informal payments also lead to extortion by health workers and bribery by the patients with the aim of getting quality treatment which they already have a right to [16]. There is psychological fear of not having access to care if no payment is made by the patient/guardian and therefore bribery by health care seekers to get what they think is better care comes into play. Unfortunately, informal payments have been normalized to appear as an act of gratitude [17], which makes informal payment malpractice difficult to tackle. Informal payment, limits financial protection [18] and may push the households into poverty undermining what the UHC (Universal Health Coverage) advocates for [19].

A Western Balkan study reported that despite evidence regarding informal payments by patients, the data are contradictory, which makes informal payments a complicated matter and therefore makes the development of tangible strategies to deter informal payments difficult [20].

Research in post-Soviet countries and Mongolia found that the poorest households were more likely to make informal payments at public facilities and that socioeconomic disadvantage was directly associated with the likelihood of patients making informal payments [21]. A sub-Saharan study found that the poorest households often delayed seeking help or did not seek help at all when ill because they could not afford to make informal payments [22]. Despite health workers being the perpetrators of malpractice, some patients give informal payments as “a gift” in seeking to obtain quality service and receive care quickly [4]. Some patients do not participate in informal payments because they believe it is not proper, and other patients cannot afford informal payments due to poverty or a lack of money. Patients who make informal payments usually *jump the queue and receive better service or more care* [11]*.*

Informal payments for health services are common in many countries globally, especially in low- and middle-income countries [23]. For instance, a Bulgarian study found that 13% of users reported making informal payments for outpatient visits and that 33% of users reported having made informal payments for hospitalization [24]. More than 50% of the sample had a negative attitude towards informal payments, but 27% of respondents had a positive attitude towards the practice. It was also reported that Albania, one of the poorest countries in Europe, provides most health care services free of charge, but informal payments are more common than in other European countries [20]. Research showed that 60–70% of Albanian citizens make informal payments to doctors to receive services and that factors influencing informal payments in Albania include low salaries of health workers, the desire to receive better quality care, the tradition of giving gifts to show gratitude and a lack of deterrents [25] to expose or stop perpetrators who indulge in this behavior.

Furthermore, in Niger’s health sector, the practice of informal payments is explained based on the sociocultural context and is considered “voluntary” behavior related to the culture of gift giving [26]. Another study in Tanzania, found that health workers at all levels receive informal payments and sometimes share payments across cadres [27].

Malawi has not been spared from this malpractice, and the authorities through the ministry of health are aware of presence of informal payments in its public facilities. This is evidenced through its communication on this malpractice [28]. There have been a few reports of informal payments in health care in Malawi [29], resulting in limited literature on the subject and hence the need to study informal payments in Malawian public hospitals. Very few studies offer a glimpse into the existence of informal payments in Malawian health care, and they have also not documented the magnitude of the problem. One of the studies found that due to low salaries, health workers are tempted to accept gifts or demand payment from patients for a service [22]. Patients are sometimes pressured to pay for services provided at the public health facility, which are officially provided for free so that they are attended to faster or receive better service [2].

Therefore this study aimed to investigate informal payments for health care in Malawi to determine the prevalence of the practice, the factors that influence it, the services that are associated with informal payments and the challenges that guardians and patients as well as the health system face as a result of informal payments. The findings may help to influence policy formulation to curb malpractice in Malawi and elsewhere.

## Methods

### Study design and setting

A cross-sectional study using a mixed methods approach was conducted at Kamuzu Central Hospital (KCH) located in the central region of Malawi between July 2017 and June 2019. KCH was chosen because it is the largest referral hospital in the central region and Lilongwe District, the capital city of Malawi. KCH provides tertiary health services to a catchment population of over four million. This study was conducted in the gynecology, surgery, outpatient and eye departments. These departments were chosen because they are highly patronized, and the researchers believed they would provide rich data.

### Study population

The study targeted patients, guardians and staff at KCH. It also included national-level key informants at the Ministry of Health, Directorate of Quality Management. The staff (hospital managers and heads of departments at KCH) were interviewed as key informants. In this study, we identified a key informant as a person whose position in a research setting gives him or her specialist knowledge about other people and processes that is more detailed and is a particularly valuable source of information to a researcher. In total, three focus group discussions (FGDs), seven key informant interviews (KIIs) and a sample size of 295 were used to conduct this study.

### Sampling and sample size determination

To calculate the sample size for the quantitative component, the study adopted a statistical formula proposed by Cochran and expanded by Yamane to calculate the sample size for this study [30]. KCH receives approximately 800 patients every day. With a 95% level of significance, the study required a sample size of 266. Adjusted for 10% nonresponse, the study required a final minimum sample size of 295 randomly recruited participants. For the qualitative component, the study had 7 key informants, who were purposively selected, and 3 FGDs. The key informants included staff personnel from KCH (hospital managers and heads of departments) and one member from the Quality Management Unit.

### Data collection and analysis

A structured questionnaire was administered to collect quantitative data, while in-depth interview and FGD guides were used to collect qualitative data. The data collection instruments were translated and piloted prior to data collection.

Quantitative data were analyzed using STATA, and the data are presented in tables and graphs. To determine the relationship between the frequency distribution of the respondents based on demographic factors and informal payments, we performed Fisher's exact test; to determine the relationship between the frequency distribution of respondents based on other variables and informal payments, we conducted the chi-square test. A Tobit regression model was also used with the truncated method to investigate factors affecting the frequency of informal payments to access health care services. An ordered regression model was used due to the ordinal pattern of the dependent variable (informal payment). Using the truncated method, we excluded observations with no informal payments from the sample.

Thematic content analysis was performed to analyze qualitative data. All FGDs and KIIs were digitally-recorded and transcribed verbatim, with the FGDs translated into English for analysis. Careful and repeated reading of the transcribed texts helped the researcher identify patterns and trends in the participant responses. The data were coded manually, and themes were inductively and deductively identified and categorized based on similarities and differences.

## Results

### Demographic characteristics of the study respondents

Table 1, indicates that a total of 298 respondents participated in this study. Among these respondents, 134 (45%) were men, while 164 (55%) were women. The table presents in detail the demographic characteristics of the respondents according to gender.

**Table 1:** Demographic characteristics of the study participants according to gender

Characteristic	Male (n = 134, 45%)	Female (n = 164, 55%)
*Age range*		
18–25 years	32 (23.9%)	33 (20.1%)
25–35 years	44 (32.8%)	47 (28.7%)
35–45 years	39 (29.1%)	39 (23.8%)
45–55 years	15 (11.2%)	28 (17.1%)
Above 55 years	4 (3.0%)	17 (10.4%)
*Marital status*		
Married	99 (73.9%)	123 (75.0%)
Divorced/separated	12 (9.0%)	5 (3.0%)
Widowed	3 (2.2%)	3 (1.8%)
Never married	20 (14.9%)	33 (20.1%)
*Occupation*		
Formal employment	38 (28.4%)	68 (41.5%)
Self-employed	50 (37.3%)	44 (26.8%)
Not employed	45 (33.6%)	52 (31.7%)
Others (specify)	1 (0.7%)	0 (0.0%)

### Informal payments according to selected demographic characteristics

Table 2 presents the median informal payments made in cash based on the demographic characteristics of the participants. There were no significant differences in the median informal payment made with respect to age (*P* = 0.022), gender (*P* = 0.4520) or marital status (*P* = 0.075), but there were significant differences by age group and occupation (*P* = 0.034).

**Table 2:** Informal payments according to the demographic characteristics of the respondents

Variable	Median (payment)	Test
*Age*		
18–25	4055	Kruskal–Wallis *P* = 0.022 Significant
25–35	7264	
35–45	4360	
45–55	12,809	
> 55	18,846	
*Gender*		
Males	12,394	Kruskal–Wallis *P* = 0.4520 Not significant
Females	5027.27	
*Marital status*		
Married	4000	Kruskal–Wallis *P* = 0.075 Not significant
Divorced/separated	600	
Widowed	25,000	
Never married	2750	
*Occupation*		
Formal employment	5000	Kruskal–Wallis *P* = 0.034 Significant
Self-employed	3000	
Not employed	3500	

Table 3 shows that the occupation and age of the patients highly influenced the probability of making informal payments for health care (correlation coefficient = 0.1168, *P*-value = 0.04; correlation coefficient =  − 0.2802; *P*-value < 0.001, respectively). However, informal payments for patient care were not statistically correlated with marital status or gender (correlation coefficient = 0.1011; *P*-value = 0.0816; correlation coefficient =  − 0.0346; *P*-value = 0.5522, respectively).

**Table 3:** Correlation coefficients between informal payments and demographic characteristics

Characteristic	Coefficient	
Payment	1	*P* = 0.0439
Occupation	0.1168	1
Payment	1	*P* = 0.0816
Marital status	0.1011	1
Payment	1	*P* = 0.5522
Gender	− 0.0346	1
Payment	1	*P* < 0.001
Age range	− 0.2802	1

### Prevalence and amounts of informal payments

The study found that 97% (240/248) of the participants knew about the existence of informal payments in public hospitals and that 47% of them had made informal payments for health services in the form of cash (Table 4). The median amount paid by the patients irrespective of age and gender was MK 600 (USD 0.83). It was also established that 87% of the informal payments were requested by health care workers while 13% were initiated by the patients themselves. Table 4 presents the results on knowledge of informal payments for health care among the clients and patients attending curative services.

**Table 4:** Payments for health services reported by patients exiting the health facility

	Male		Female	
*N*				
	134		164	
	Yes	No	Yes	No
	% (95% CI)	% (95% CI)	% (95% CI)	% (95% CI)
Knowledge of payment for services	110(74–87)	24(13–25)	131(72–85)	33(14–27)
Of those who paid for services received, the median amount paid (MK):				
*N*	Median (IQR)		Median (IQR)	
	62		66	
Services paid for	1000(2250.00–5000.00)		600(2000.00–6000.00)	
N	52		67	
	Health care worker	Patient	Health care worker	Patient
	% (95% CI)	% (95% CI)	% (95% CI)	% (95% CI)
Who initiated the informal payment	87(74–94)	13(07–25)	87(75–92)	13(07–25)

Lastly, Table 5 (Tobit regression model) shows that in accordance with prior expectations and theoretical predictions, all occupation categories and gender were significantly associated with informal payments, although informal payments from individuals who were self-employed or not employed were slightly lower than those from individuals who were employed. All the results were significant at *P* < 0.05. There was no association between being married and making an informal payment, *P*-value > 0.05. Male gender was associated with a lower number of informal payments offered than female gender.

**Table 5:** Determinants of informal payments using the Tobit regression model

Characteristics	Informal payment in cash initiated by the client		Informal payment in cash initiated by the health care worker	
Variables	β	(SE)	β	(SE)
*Gender (Ref: Female)*				
Male	− 8624.096*	1633.17	− 3705.45	1861.90
*Marital status (Ref: married)*				
Divorced/separated	3863.07	3199.02	5350.42	3919.59
Widowed	14,084.74	8872.56	–	–
Never married	645.23	2538.07	− 1153.66	2837.98
*Age group (Ref: 18 to 25 years)*				
Above 25 to 35 years	1022.65	3304.85	1140.85	3761.68
Above 35 to 45 years	429.59	3373.28	– 175.33	3854.58
Above 45 to 55 years	7391.60	3670.62	6861.87	4159.00
Above 55 years	7010.35	3908.23	9006.57	4989.25
*Occupation (Ref: formal employment)*				
Self-employed	− 4943.29*	1819.99	− 5908.32*	2030.74
Not employed	− 4686.75*	2188.09	− 4798.52	2679.21

*P < 0.05; **P < 0.01; ***P < 0.001

From a qualitative perspective, some guardians indicated that they were aware that informal payments were practiced at KCH for patients to access some health care services. Others indicated that they had never heard about the practice or experienced it. The guardians who acknowledged the existence of informal payments asserted the following:

"*…what surprised me is that after an operation, I was asked to pay to get the results; I paid K9000 ($12). So, I asked them, why they didn’t tell me in the first place that I would have to pay? [Because] I didn’t want to argue with them, I was angry, and I just paid K9000 and left for home.”* (FGD 3)

The guardians who had never experienced or heard about informal payments at KCH indicated that if they were asked to pay some money to ensure treatment for their relations, they would pay to save their life:

“*I can pay, if I have the money. This is a difficult situation because you can’t just sit and see your child in pain; you can even sell a cloth so that you find money and can pay the doctor so that your child will be saved*." (FGD 2)**.**

Majority of key informants indicated that they were aware of informal payment practices at KCH. Most of them stated that they had evidence of informal payments, while a few indicated they had just heard about it but had no evidence. One of the participants said,

"…. *because there was a long queue [and] a lot of people were waiting, the man who sorts the healthy passports tipped my father that ‘if you want to be assisted quickly, you should give me something.’ Then, because my father was desperate …. [and] when I was getting there, my father said he was happy [because]… ‘you know I have been assisted very quickly because this guy asked me to give him money so that he would give the one who operates the X-ray to do it faster.*" (Key informant 4: Nurse)

### Factors influencing patients’ decisions to make informal payments

Different patients had different purposes for making informal payments. The study found that 54% of patients paid because they were seeking to receive holistic care, 25% paid just to express their gratitude, and only 11% paid because they wanted quick services. Of these payments, 2% were made before the services were provided, 5% were made during the provision of services and 13% were made after the services were provided.

### Fear and lack of knowledge

Some guardians and patients were compelled to pay money for services that are formally offered for free out of fear of not being treated by doctors, which could result in the death of their loved ones. This situation was also aggravated by a lack of knowledge by the patients and guardians about what to do when health care providers demand payment for services that are formally offered for free:

“*Fear makes us pay. We are afraid that if we don’t do what we are being asked to do, we will lose our beloved because they will not be treated, and because of that, we are forced to pay. Sometimes one can pay because maybe she or he does not know how things work. But the bottom line is that they fear they might lose their beloved if they don’t pay (because their patient will not be treated)*.” (FGD 3)

### Desperation

The guardians stated that illness makes people desperate, such as when patients and guardians arrive at the hospital and they are willing to do anything possible, including making informal payments to see that their loved one receives treatment:

“*This is a difficult situation because you can’t just sit and watch your child who is in pain. You can even sell a cloth to raise money and pay the doctor so that your child is saved*.” (FGD_2)

This statement corroborates what the key informants said about serious illnesses forcing patients to informally pay health care workers so that they are treated quickly to prevent the patient’s condition from deteriorating:

“*A good example was, I met one man who was so desperate [because] his mother had a tumor in the brain, and they (the doctors) were saying the earlier it was removed, the better, before it degenerated into cancer. So he (the guardian) went and looked for the money and paid*”. (Key informant 3)

### Types or modes of informal payments

**Fig. 1:**
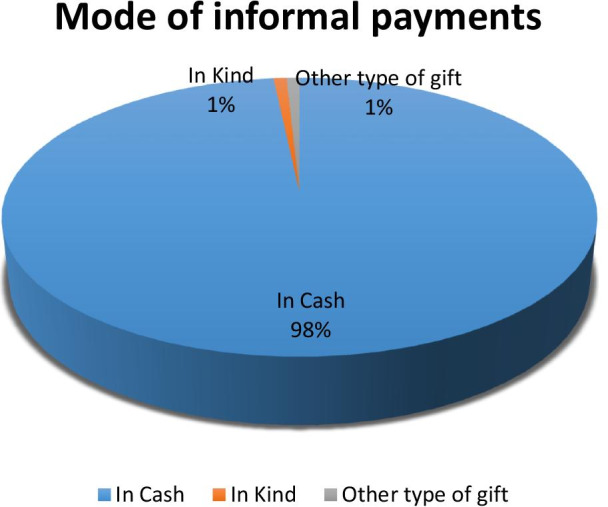
This figure presents the types/modes of informal payments and frequency of informal payments that were made by the patients

**Fig. 2:**
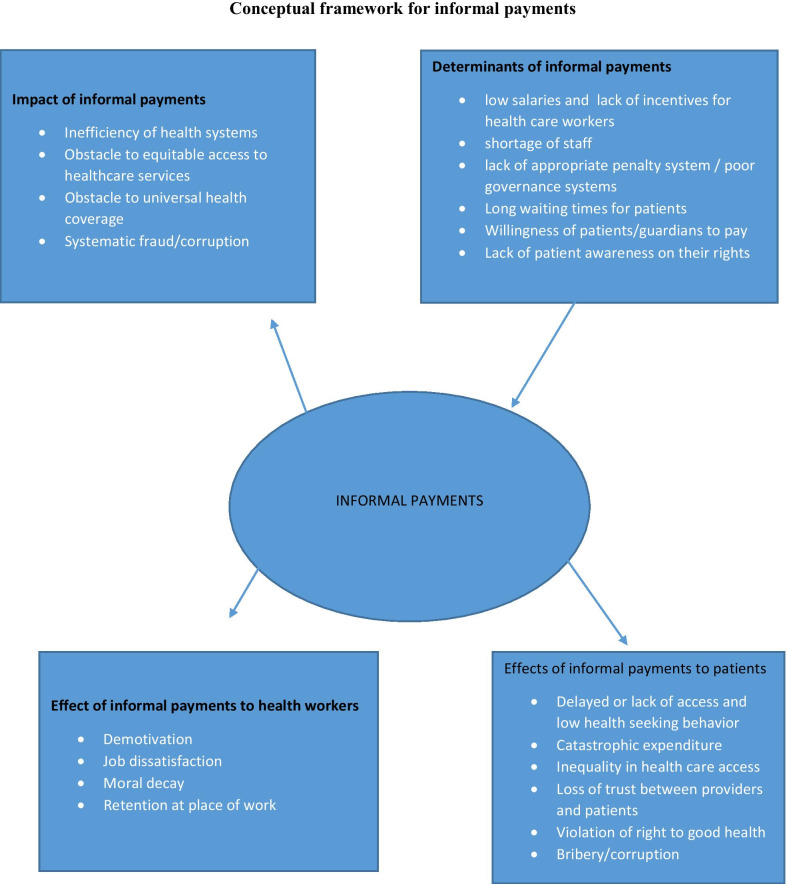
Presents a conceptual framework showing impact, determinants of informal payments and effects of informal payments on both health care workers and patients/guardians

This section presents the types/modes of informal payments and frequency of informal payments that were made by the patients. It was found that most (98%) of the informal payments were made in cash and were made to the hospital staff to ensure better services and Fig. 1 shows the valid percentage of participants who reported making informal payments in cash or in kind (Fig. 2).

### Factors influencing health workers to demand or accept informal payments

#### Self-indulgence

Participants in all categories suggested that health care providers were not justified to demand informal payments from patients and guardians but rather their desire to amass more money pushed them into this practice. They felt that since there were other health care workers who did not get involved in informal payments, therefore those who did, did it because they lacked a moral compass:

“*Some health care providers are merely greedy because even those who are financially stable still demand or accept money from patients*.” (Key informant: Doctor 1)

#### Shortage of resources: drugs and staff work overload (higher demand than supply)

Participants consistently indicated that shortages of resources such as drugs, equipment and human resources contributed to informal payment practices. This imbalance led to more waiting time; as a result, the patients or guardians resorted to bribing health care workers to fast-track access to health care services. In addition, due to a shortage of staff, health care workers felt they were overworked for less pay and resorted to demanding or accepting money or materials from patients as compensation:

“*KCH serves a big population of people but is understaffed. There are always a lot of patients, and most want to be assisted quickly and go to attend to other matters. Because these people are desperate to get quick service, they give money to the health worker. [On the other hand], health workers feel they are not compensated fairly, and they start demanding money from patients.”* (Key informant 5: Human resource staff)

"*And these other problems arise from a shortage of medicine in hospitals, and since the medicine is not enough, the health workers start to help their relations or friends only. If you are not related to him (the health worker), you don’t get treatment, and if you also don’t have money to bribe the doctor, you don’t get treatment*." (FGD 2)

#### Low salaries

Some health care providers, such as patient attendants and cleaners, demanded or accepted money from patients because of their low salaries:

*"I know, for example, in the orthopedic department, there are patient attendants; they work hard, but you will hear that they get K50000 or K40 000 (US$55-69) a month. These people have to pay rent and send their children to school. When they see that their seniors are receiving money from the patients, you know, they also do the same. And I understand they are doing this because they don’t get paid enough money*." (Key informant 1: Doctor)

#### Lack of effective disciplinary measures

There is a lack of effective punishments for offenders, which encourages health care providers to demand payment for services that are formally offered for free. The health care providers know that nothing will happen to them even if they are caught accepting money from patients:

“*Loopholes in disciplinary actions make health workers comfortable continuing indulging in this bad behavior. There are no repercussions for the offender.*” (Key informant 5: Human resource manager)

## Discussion

This study revealed that people have knowledge about the existence of informal payments in public hospitals and perceive them to be unethical because they are a form of corruption that hinders poor people from accessing health services. Even though some participants stated that they would refuse to pay for a service they knew to be provided for free to discourage corruption, it is our view that such refusal would not be easy because it was illustrated that informal payments were mostly made out of desperation to receive service. Similar sentiments have been expressed in studies in other countries. For example, a multicountry study in Albania, Bulgaria, Poland and Lithuania indicated that 64% of the participants indicated they would rather go to a private hospital than make informal payments in public hospitals for services that should be offered for free [25].

There are a number of factors that were identified in both the quantitative and qualitative studies, including low salaries, shortage of staff, and lack of strict disciplinary actions against members of staff who engage in informal payments. These findings are consistent with studies done elsewhere, for example a study done in Albania identified low salaries for health workers and weak disciplinary measures to punish offenders as some of the key factors contributing to informal payments in public health facilities [25].

Most key informants thought that informal payments were a form of gift and therefore not harmful. However, when asked about the underlying causes of out-of-pocket payments, they concurred with what our study found, i.e., that low salaries are the driving factor for informal payment [25]. In Romania, providers also described informal payments as gifts/presents (chocolate, flowers, cash etc.) [31]. A Bulgarian study found similar results and reported that informal payments continued to be made because of poor adherence to law by both citizens and government officials and the lack of governmental effort to increase salaries and generally increase funding for health care [32]. Hence, the government needs to improve the salaries of workers to address informal payment issues.

In both the qualitative and quantitative results, the views of the participants corroborated the assertion that a lack of effective punishments for offenders encourages health care providers to demand payment for services that should be offered for free. The Malawian health system has good public services regulations, but they are never applied when a situation arises. As a result, people involved in malpractice are not punished due to a lack of evidence or favoritism for those who have relations and friends at the ministry headquarter. Nodeh et al. [33] recommended enforcing rules and punishing offenders, and Malawi could follow this approach. Emphasis should be placed on formulating deliberate policies and rules prohibiting informal payments to deter abuses [33].

If patients had reasonable waiting times to access health care, guardians or patients would not be forced to pay. These sentiments were evidenced in both the qualitative and quantitative data, which indicated that when guardians spend days or weeks in the hospital, they feel they are being delayed and thus resort to making informal payments for the services so that they can return home quickly. A Nigerian study, reported similar findings, i.e., that in some cases, patients make informal payments to jump the queue and receive better quality services or more care, thereby limiting access to health services to patients who have greater ability to pay rather than those most in need [34].

Illness makes people desperate; as such, when the patients and guardians arrive at the hospital, they are willing to do anything possible, including making informal payments, to see their loved ones receive the needed health services. This outcome is consistent with Moldovan’s study, which indicated that patients felt they had to pay as demanded, “because you have to give it. Otherwise, you won’t receive the service you need” [32].

The study reported that there was a lack of knowledge on the part of the patients and guardians about what to do when health care providers demand payment for a service that should be free. Most patients in public hospitals do not know their rights and responsibilities when accessing care, which, coupled with a lack of an assertive nature, accelerates malpractice; most patients fail to demand free services and question anything other than free services. The good news is that a hospital ombudsman office has been recently set up by the Ministry of Health in health facilities to address complaints and grievances of those accessing health services.

The blame for informal payments should not entirely rest on health workers but also patients/guardians because most studies, including this study, established that both patients and health workers initiate informal payments at some point [24]. This result shows that it will take both community members and health workers to control or end informal payment practices.

Informal payments results in loss of possessions and poor health-seeking behavior among patients and guardians. Some respondents in this study reported that when they paid money for health services, they were left with no means to buy food or even pay for transport to return home when patients were discharged. These results are similar to those of a Bulgarian study that found that among individuals who paid for health services, approximately 6% borrowed money to pay for services, and more than 10% of users borrowed money to pay for hospitalization [11]. In addition, 32% of the sample forewent physician visits due to their inability to make informal payments. It can be concluded, therefore, that the practice of informal payments negatively affects the health-seeking behavior of the public despite government efforts to promote universal access to health.

There was a feeling of regret from the key informants (senior hospital staff), who suggested that informal payments have generally created a negative reputation for hospitals and health workers, as people think that every health care worker at KCH is involved in malpractice. Malawi depends on the donor community’s support to sustain its health sector; therefore, informal payments will likely destroy its credibility and trust from development partners who are essential in supporting government social service delivery [35].

The study findings are consistent with the findings of Schaaf and Topp [8] who reported that informal payment practice has created inequalities in accessing care between those who have money and those who do not have money to pay for services when health care services should be provided for free [8]. Health care workers who accept informal payments prioritize patients who have paid money, leaving out those who cannot afford to pay, therefore creating inequalities in accessing health care services.

It is challenging to end informal payments because health care providers involved in this practice often walk free since management sometimes does not discipline the perpetrators because they also benefit from the payments. Additionally, the Malawian culture of silence makes patients afraid to report or speak out against informal payments and this contributes to the increase in this malpractice. Qualitative results indicate that since this practice is shrouded in secrecy, it is hard to end it because patients and guardians are afraid to talk about it openly due to fear of unknown consequences from the perpetrators.

## Conclusion

Informal payments in public hospitals are an issue of concern in Malawi, as reported in this study. The key factors contributing to informal payments include understaffing, low salaries and lack of incentives for health workers and lack of disciplinary measures for offenders. Therefore, there is a need for policy makers and health administrators to develop policies and regulations with clearly defined, strict disciplinary actions to be taken against the perpetrators of informal payments. Another recommendation is that the government should improve the working conditions for health workers so that they are not tempted to extort money from patients. Furthermore, patients and guardians should be made aware of their rights and responsibilities in accessing health care services so that they are able to demand accountability from health care services and report requests to pay for a service they know should be accessed for free.

Lastly, it should be mentioned that locating participants was a challenge, as some potential participants were unwilling to provide sensitive and accurate information due to the sensitivity of the topic of study. Time and resource constraints were also limiting factors and forced the researcher to conduct the study only at KCH instead of all the central/referral hospitals in Malawi.

## Data Availability

Data and materials supporting the conclusions used in the manuscript are available from the corresponding author on request.

## Abbreviations

KCH: Kamuzu Central Hospital
SDGs: Sustainable development goals
OOP: Out of pocket
KII: Key informant interview
FGD: Focus group discussion
HIV: Human immunodeficiency virus
UHC: Universal health coverage
HSSP: Health sector strategic plan
EHP: Essential health package
WHO: World Health Organization
